# Fusarium keratitis in an older adult without exogenous or traumatic risk factors: A case report on intrinsic ocular surface predisposition

**DOI:** 10.1016/j.idcr.2026.e02652

**Published:** 2026-06-25

**Authors:** Tingting Huang, Guodong Liang, Xi Jiang, Yuxing Gao, Huiyi Wu

**Affiliations:** a Department of Clinical Laboratory, Affiliated Donghai Hospital of Kangda College of Nanjing Medical University, Donghai County People's Hospital, Lianyungang, Jiangsu 222300, China; b Department of Blood Transfusion, Affiliated Donghai Hospital of Kangda College of Nanjing Medical University, Donghai County People's Hospital, Lianyungang, Jiangsu 222300, China

**Keywords:** Fusarium keratitis, Fusarium solani species complex (FSSC), MALDI-TOF MS, Older adult, Ocular surface, Case report

## Abstract

**Objective:**

To report a case of Fusarium keratitis in an 80-year-old female without typical exogenous or traumatic risk factors, such as ocular trauma, contact lens use, or previous ocular surgery, but with intrinsic ocular surface predisposing factors, and to highlight the diagnostic value of MALDI-TOF MS.

**Methods:**

Routine bacterial and fungal cultures were performed on ocular secretion samples from the patient. Lactophenol cotton blue staining was used for microscopic examination, and MALDI-TOF MS (Matrix-Assisted Laser Desorption/Ionization-Time of Flight Mass Spectrometry) was employed for rapid and accurate identification of the fungal strain. Clinical data were comprehensively analyzed.

**Results:**

White, cotton-like fungal colonies were observed on blood agar and chocolate agar plates. Microscopic examination revealed transparent, septate hyphae and numerous long, oval conidia, initially suggesting Fusarium spp. MALDI-TOF MS analysis showed a high-confidence match with *Fusarium solani*, and the isolate was interpreted as *F. solani* / *Fusarium solani* species complex (FSSC) based on morphology and MALDI-TOF MS.

**Conclusion:**

This study highlights the clinical value of MALDI-TOF MS in the identification of pathogens in complex ocular infections. Despite the absence of typical exogenous or traumatic risk factors, the patient developed rapidly progressive invasive keratitis. This case suggests that intrinsic ocular surface abnormalities in older adults, including tear film instability, epithelial barrier impairment, and corneal nerve degeneration, may lower the threshold for fungal infection. The possible contribution of immunosenescence should be interpreted only as contextual background and remains to be validated in future studies.

## 1 Introduction

Fungal keratitis (FK) is one of the leading infectious causes of corneal blindness globally, with over one million new cases reported annually. This condition places a substantial burden on both visual health and socio-economic systems [1]. The incidence of FK is notably higher in tropical and subtropical regions, with *Fusarium* species emerging as the predominant pathogenic fungi. Multi-center studies have demonstrated that *Fusarium* accounts for 40–60% of FK cases, with some regions reporting even higher prevalence rates [2], [3], [4]. Although substantial epidemiological data exist, there remains a paucity of clinical evidence regarding the heightened susceptibility of older adults individuals to ocular surface infections due to immune senescence.

While Fusarium keratitis is typically associated with exogenous or traumatic risk factors such as plant-related ocular trauma, contact lens use, or a history of ocular surgery, this case presents a rare clinical scenario of an older adult without these external triggers but with significant intrinsic ocular surface abnormalities, suggesting that local ocular surface compromise may have contributed to susceptibility. Diagnosis of FK generally relies on clinical presentation, culture, and morphological observation; however, these methods are often time-consuming, limiting the opportunity for early intervention. In recent years, matrix-assisted laser desorption/ionization time-of-flight mass spectrometry (MALDI-TOF MS) has emerged as a promising tool for rapid pathogen identification, facilitating the detection of both bacterial and fungal pathogens. However, further prospective clinical validation is needed for its application in FK [5], [6], [7].

The rapid aging of the global population has led to an increased prevalence of older adults individuals, a group that remains underappreciated as a high-risk population for FK. Previous studies suggest that older adults may experience reduced tear secretion, impaired tear film stability, and compromised ocular surface barrier function, which may increase susceptibility to ocular infection. However, the role of immunosenescence in fungal keratitis remains incompletely defined and requires further clinical validation [8], [9], [10]. Epidemiological studies indicate that 70–90% of *Fusarium solani*-induced keratitis cases are associated with a clear history of ocular trauma or contact, while non-traumatic cases are relatively rare, often accounting for less than 5–10% of cases. These non-traumatic cases tend to occur in patients with immune dysfunction or tear film abnormalities.

This study reports a case of an 80-year-old female patient who developed rapidly progressive invasive Fusarium keratitis in the absence of typical exogenous or traumatic risk factors. Importantly, the patient had several intrinsic ocular surface predisposing factors, including reduced tear secretion, tear film instability, increased tear osmolarity, and corneal nerve degeneration. Using MALDI-TOF MS technology, the pathogen was rapidly and accurately identified. In addition, the case discusses possible intrinsic ocular surface predisposing factors and briefly considers immunosenescence as a literature-supported contextual hypothesis.

## 2 Case information

### 2.1 Medical history and clinical manifestations

An 80-year-old female patient was admitted with a history of "redness, swelling, pain, and decreased vision in the left eye for more than one month." One month prior to admission, the patient reported a mild foreign body sensation in the left eye, without any history of trauma or exposure to agricultural materials. The patient self-administered levofloxacin eye drops for approximately 5 days; however, her symptoms did not improve and progressively worsened. One week prior to hospitalization, the patient experienced significant eye pain, tearing, photophobia, and a marked deterioration in visual clarity. She denied any history of wearing contact lenses, ocular surgery, or diabetes. However, the patient had clinically relevant intrinsic ocular surface abnormalities, including a predisposition to dry eye, reduced tear secretion, tear film instability, and reduced corneal sensation, which may have compromised the local ocular surface defense. The patient had a history of age-related cataracts and a predisposition to dry eyes, for which she had been using artificial tears regularly, but denied any chronic corneal diseases.

On examination, visual acuity in the left eye was limited to hand movements in front of the eye. The eyelid exhibited mild edema, and there was marked conjunctival injection (+++). A 5-mm diameter grayish-white infiltrate was observed in the central cornea, with raised, dry surfaces and feathery margins, accompanied by satellite lesions and approximately 2 mm of hypopyon in the anterior chamber. Corneal sensation was significantly reduced. The right eye showed a visual acuity of 0.6 without abnormalities.

Considering the patient’s advanced age, the absence of a clear history of trauma, the rapid progression of infection, and the failure to respond to antibiotic therapy, the provisional diagnosis was "left-eye fungal keratitis." A sample was immediately sent for pathogen testing.

### 2.2 Ocular surface microenvironment evaluation

To further assess the patient’s ocular surface barrier function, additional tests were performed to evaluate tear film secretion, tear film stability, and corneal nerve health:

#### 2.2.1 Schirmer I test

Left eye: 4 mm/5 min, Right eye: 6 mm/5 min (normal value >10 mm/5 min).

The results indicated significantly reduced tear secretion, consistent with age-related dry eye syndrome.

#### 2.2.2 Tear break-up time (BUT)

Left eye: 4.2 s, Right eye: 5.1 s (normal value >10 s).

The reduced BUT suggests a decrease in tear film stability, a critical factor contributing to corneal exposure and epithelial vulnerability.

#### 2.2.3 Tear osmolarity

TearLab® test results: Left eye: 316 mOsm/L, Right eye: 308 mOsm/L (≥308 mOsm/L is indicative of dry eye).

The elevated osmolarity in the left eye is consistent with increased tear film inflammation and a heightened risk of epithelial damage.

#### 2.2.4 Corneal nerve structure assessment (IVCM)

Confocal microscopy of the cornea revealed reduced nerve fiber density in the superficial stromal layer of the left eye, with thinning and fragmentation of the fibers. A mild reduction was noted in the right eye.

This change suggests age-related degeneration of the corneal nerves, which may lead to diminished corneal sensitivity and impaired epithelial repair capabilities.

These findings indicate that although the patient lacked typical exogenous or traumatic risk factors, she had significant intrinsic ocular surface predisposing factors. Reduced tear secretion, tear film instability, increased tear osmolarity, and corneal nerve degeneration may have compromised epithelial barrier integrity and local defense, thereby contributing to increased susceptibility to Fusarium infection.

### 2.3 Laboratory tests

Peripheral blood routine and inflammatory markers were assessed. The results indicated a white blood cell count of 6.8 × 10⁹/L, with 68% neutrophils. C-reactive protein (CRP) was 8.2 mg/L, and procalcitonin (PCT) was 0.06 ng/mL, with no significant signs of systemic inflammation. Blood glucose, liver and kidney function, and immunoglobulin levels were all within normal reference ranges.

### 2.4 Microbiological identification

#### 2.4.1 Culture and morphological observation

##### 2.4.1.1 Culture media

The ocular secretion samples were simultaneously inoculated onto blood agar and chocolate agar plates.

##### 2.4.1.2 Incubation conditions

The inoculated plates were incubated at 25°C in a 5–10% CO₂ atmosphere.

##### 2.4.1.3 Colony morphology

After 24–72 h of incubation, colony characteristics were observed. The colonies exhibited typical *Fusarium* features: initially appearing as white, fluffy colonies, they rapidly expanded into white, cotton-like colonies with abundant aerial mycelia.

#### 2.4.2 Microscopic identification

**Fig. 1:**
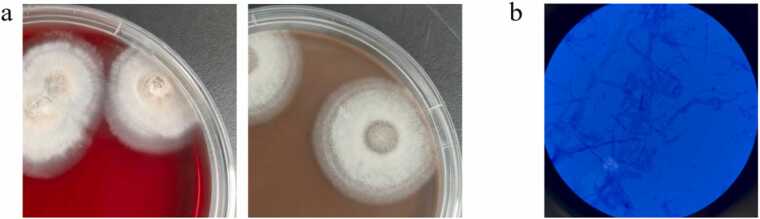
Colony Morphology and Micromorphological Features of the *Fusarium solani* / FSSC Isolate. a: Colony growth on blood agar and chocolate agar plates, showing white, cotton-like colonies with abundant aerial mycelia. b: Lactophenol cotton blue staining showing hyaline septate hyphae, phialide-like conidiogenous cells, and smooth oval-to-cylindrical microconidia. Typical canoe- or sickle-shaped macroconidia and chlamydospores were not clearly demonstrated in this microscopic field.

Pure colonies were selected for lactophenol cotton blue staining. Microscopic examination revealed hyaline, septate hyphae and phialide-like conidiogenous cells, accompanied by numerous smooth, hyaline, oval-to-cylindrical microconidia. However, typical canoe- or sickle-shaped macroconidia and chlamydospores were not clearly demonstrated in the examined microscopic field. Therefore, the morphological findings supported identification as *Fusarium* spp., but were insufficient alone for definitive species-level identification. The final identification was based on the combined results of colony morphology, micromorphology, and MALDI-TOF MS (The results are shown in Fig. 1).

#### 2.4.3 MALDI-TOF MS spectral identification

The pure culture was analyzed using MALDI-TOF MS. The procedure was as follows: a single colony, cultured for 48 h, was selected and directly smeared onto the surface of a MALDI-TOF MS target plate. After drying, 1 μL of α-cyano−4-hydroxycinnamic acid (HCCA) matrix solution was added. Detection was performed using the Autof ms1000 automated microbiological mass spectrometry system (Autobio Diagnostics, China). It should be noted that MALDI-TOF MS analysis was performed after sufficient fungal colony growth had been obtained from culture. Therefore, the approximately 15-minute interval referred only to the analytical phase, including colony transfer, matrix application, spectral acquisition, and database matching, rather than the total diagnostic turnaround time from specimen collection. The mass spectrometry database contains over 10,000 bacterial and fungal standard spectra, covering more than 120 clinically relevant filamentous fungi, enabling genus-level and, in most cases, species-level identification.

**Fig. 2:**
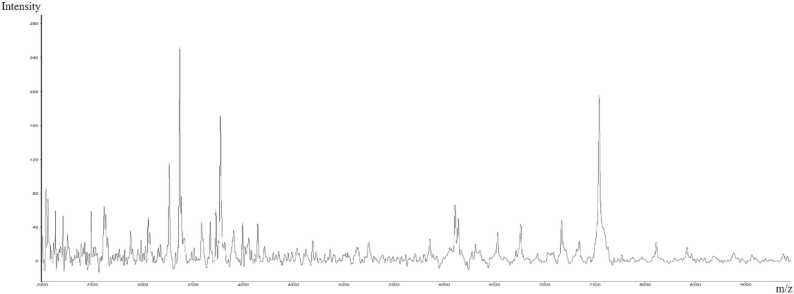
MALDI-TOF MS Protein Fingerprint of the Isolate Showing a High Match with *Fusarium solani* / FSSC in the Database.

The results showed that the protein fingerprint from the mass spectrometry analysis matched *Fusarium solani* in the database with a confidence score greater than 2.300 (Fig. 2), exceeding the species-level identification threshold of 2.000. Together with the morphological findings, the isolate was identified as *Fusarium solani* / *Fusarium solani* species complex (FSSC). However, because *F. solani* belongs to the FSSC, accurate differentiation of closely related species within this complex generally requires molecular sequencing methods, such as internal transcribed spacer (ITS), translation elongation factor 1-alpha (TEF1-α), or β-tubulin gene analysis. Since molecular sequencing was not performed in this case, the identification should be interpreted as *F. solani* / FSSC based on morphology and MALDI-TOF MS, rather than as definitive molecular confirmation of a specific species within the complex.

To more clearly illustrate the advantages of MALDI-TOF MS in pathogen diagnosis, a comparison was made with traditional morphological identification methods (Table 1).

**Table 1:** Comparison Between Morphological Identification and MALDI-TOF MS in Pathogen Recognition.

**Identification Method**	**Analytical Time After Colony Availability**	**Identification Level**	**Accuracy**	**Operator Dependency a**	**Clinical Application Value**
Morphological Identification	24–120 h depending on colony growth and morphological maturation	Primarily genus level, some species level	Moderate (∼80%)	High (Score: 4/5)	Long turnaround time, experience-dependent
MALDI-TOF MS	15–30 min after colony growth; total turnaround time depends on culture incubation	Genus and species level	High (>95%)	Low (Score: 1/5)	Rapid, accurate, significantly reduces diagnostic time

Note: For MALDI-TOF MS, the 15–30-minute interval refers only to the analytical phase after sufficient fungal colony growth and does not include the preceding culture incubation period. Therefore, the total diagnostic turnaround time remains dependent on fungal growth and colony availability. For filamentous fungi, especially the *Fusarium solani* species complex (FSSC), MALDI-TOF MS may not reliably distinguish closely related species within the complex; molecular sequencing methods such as ITS, TEF1-α, or β-tubulin analysis may be required for definitive species-level identification.

The system’s automatic comparison resulted in a score of 2.304, exceeding the species-level identification threshold (≥2.000). This result, together with morphological findings, supported identification of the isolate as ***Fusarium solani*** / FSSC. However, MALDI-TOF MS does not always reliably distinguish closely related species within the FSSC without molecular sequencing confirmation. The reported analytical time refers to the MALDI-TOF MS procedure after colony growth and does not include the preceding fungal culture incubation period.

#### 2.4.4 Antifungal susceptibility testing

The isolated strain was subjected to in vitro antifungal susceptibility testing using the broth microdilution method, in accordance with CLSI M38-A3 guidelines. The results indicated the following minimum inhibitory concentrations (MICs): voriconazole 0.25 μg/mL, amphotericin B 1 μg/mL, and itraconazole 0.5 μg/mL. These MIC results showed a relatively low MIC for voriconazole, which supported the clinical selection of voriconazole-based antifungal therapy. However, because established clinical breakpoints for Fusarium species are lacking, these findings should be interpreted in conjunction with clinical response and available PK/PD evidence.

### 2.5 Treatment and outcome

Based on the MALDI-TOF MS identification and antifungal susceptibility testing results, which confirmed the strain's sensitivity to voriconazole (MIC = 0.25 μg/mL), the clinical team promptly initiated targeted antifungal therapy with voriconazole as the primary agent. The treatment regimen included local administration of 1% voriconazole combined with low-dose amphotericin B, along with oral voriconazole to achieve higher drug concentrations in the deeper layers of the cornea and aqueous humor. This approach was consistent with the antifungal susceptibility results and effectively addressed the key pathogenic features of *Fusarium solani*.

Following five days of treatment, the patient reported significant relief from eye pain, and the anterior chamber hypopyon had completely resolved. Upon follow-up at two weeks, the corneal infiltration had reduced in size from 5.0 mm to 3.2 mm. At one-month follow-up, the lesion had almost fully absorbed, leaving only a localized corneal scar. The patient's visual acuity improved to counting fingers at 30 cm, with no recurrence of the infection. The treatment response was highly consistent with the susceptibility results, highlighting the critical role of rapid pathogen identification in improving the prognosis of fungal keratitis.

It is noteworthy that the patient initially self-administered levofloxacin eye drops for approximately five days during the early stages of her illness, but the condition continued to deteriorate. Fluoroquinolone antibiotics lack antifungal activity against filamentous fungi and are unable to inhibit fungal invasion in the corneal epithelium and stroma. This can lead to symptom masking and diagnostic delays [11]. Delayed initiation of appropriate antifungal treatment allows the fungus to form deeper hyphal structures, worsening tissue damage and increasing the risk of corneal scarring and blindness [12]. In older adults, reduced tear film stability, impaired epithelial repair, and corneal nerve degeneration may compromise ocular surface defense. In this case, these abnormalities may have contributed to delayed recognition and progression of infection, although no direct immunological testing was performed [13].

This case underscores the importance of considering fungal etiology in older adults patients with keratitis that does not respond to antibiotics. Timely pathogen identification and early intervention are essential to avoid missing the optimal window for effective treatment (The results are shown in Table 2).

**Table 2:** Changes in Lesion Size and Transparency Before and After Treatment.

**Timepoint**	**Maximum Corneal Lesion Diameter / Residual Finding**	**Visual Analog Scale (VAS)**	**Lesion Change/Transparency Improvement**	**Remarks**
**On Admission**	5.0	8	No improvement in transparency	Visual acuity limited to hand motion/eye level, hypopyon present, symptoms unresolved
**5 Days After Treatment**	3.2	2	Lesion size reduced, partial transparency improvement	Pain relief, hypopyon resolved
**Follow-up at 2 Weeks**	2.0	1	Significant transparency recovery	Localized corneal scarring, visual acuity improved to counting fingers at 30 cm
**1 Month Follow-up**	Mild scarring	-	Near-complete transparency restoration	Visual acuity improved; no recurrence observed, no recurrence observed

## 3 Discussion

This case demonstrates the clinical value of MALDI-TOF MS in the diagnosis of ocular fungal infection and highlights the importance of recognizing intrinsic ocular surface abnormalities as predisposing factors for Fusarium keratitis in older adults. A key feature of this case is that the patient lacked typical exogenous or traumatic risk factors, such as ocular trauma, agricultural exposure, contact lens use, or previous ocular surgery, but had significant intrinsic ocular surface abnormalities. These included severe dry eye disease, tear film instability, increased tear osmolarity, reduced corneal sensation, and corneal nerve degeneration. Therefore, this case should not be interpreted as Fusarium keratitis occurring in the absence of all risk factors, but rather as infection occurring in the absence of external triggers in the presence of intrinsic ocular surface predisposition (The results are shown Table 3).

**Table 3:** Treatment Timeline and Clinical Outcome.

**Timepoint**	**Treatment Regimen**	**Corneal Findings**	**Lesion Change/Treatment Response**	**Remarks**
**Day of Admission**	Initiate voriconazole eye drops + oral therapy	5 mm infiltration, hypopyon	Prominent lesion, worsening symptoms	Treatment plan adjusted based on MALDI-TOF MS results
**Day 5**	Continue same regimen	Lesion reduced to 3.2 mm	Pain relief, partial transparency improvement	Significant reduction in lesion size, hypopyon resolved
**Day 14**	Continue voriconazole therapy, oral intake	Lesion reduced to 2.0 mm	Significant recovery in transparency	Corneal transparency restored, visual acuity improved to counting fingers at 30 cm
**1 Month Follow-up**	Discontinue treatment	Localized scarring	Near-complete transparency restoration	Visual acuity restored, no recurrence observed

First, this case highlights the advantages of MALDI-TOF MS in diagnosing acute ocular infections. Traditional culture and morphology-based identification require sufficient fungal growth and morphological maturation, which may take several days. MALDI-TOF MS can shorten the analytical phase once a pure fungal colony is available, with spectral acquisition and database matching generally completed within approximately 15–30 min. However, this interval does not include the preceding culture incubation period; therefore, the total diagnostic turnaround time remains dependent on fungal growth and colony availability【5,7】. MALDI-TOF MS may be clinically valuable in laboratories with appropriate instrumentation, validated fungal spectral databases, trained personnel, and quality control systems. Therefore, its utility depends on local laboratory infrastructure and implementation capacity rather than being universally applicable to all county-level hospitals**,** which is crucial for the timely initiation of targeted antifungal therapy. The timely antifungal treatment in this case was directly facilitated by the rapid diagnosis provided by MALDI-TOF MS, further validating its clinical value.

The uniqueness of this case lies in the combination of “advanced age + absence of exogenous or traumatic triggers + intrinsic ocular surface barrier dysfunction." In typical Fusarium keratitis, trauma is the most common external trigger; however, the patient in this case had no history of trauma, contact lens use, or ocular surgery. Nevertheless, she had important intrinsic predisposing factors, including tear film instability, epithelial vulnerability, and corneal nerve degeneration. These findings suggest that ocular surface microenvironmental impairment, rather than the absence of all risk factors, may have contributed to the development and progression of infection. Clinically, this emphasizes the need to evaluate dry eye disease, tear film instability, corneal sensation, and corneal nerve status when assessing fungal keratitis risk in older adults without obvious external triggers.

The peripheral inflammatory markers were mildly elevated, which is consistent with the typical clinical presentation of filamentous fungal keratitis, characterized by local tissue damage with minimal systemic inflammation【10】. This phenotype may lead to misdiagnosis or delayed diagnosis, ultimately resulting in delays in targeted treatment. In this case, the absence of trauma, contact lens use, and ocular surgery initially made the diagnosis less straightforward; however, the presence of severe ocular surface abnormalities provided a clinically plausible explanation for increased susceptibility.

From a mechanistic perspective, the findings in this case should be interpreted cautiously. The patient had objective evidence of ocular surface compromise, including reduced tear secretion, shortened tear break-up time, elevated tear osmolarity, reduced corneal sensation, and corneal nerve degeneration. These abnormalities may weaken tear film stability, epithelial barrier integrity, and local defense capacity, thereby creating a favorable environment for fungal adhesion and invasion. Although previous studies have suggested that aging may be associated with impaired ocular surface immunity, delayed epithelial repair, altered tear film antimicrobial components, and dysregulated mucosal immune responses, no immunological, histopathological, or molecular investigations were performed in this patient. Therefore, the possible contribution of immunosenescence in this case should be regarded only as a literature-supported contextual hypothesis rather than a proven causal mechanism.

In this case, the failure of antibiotic treatment and the continued progression of the lesion are consistent with the clinical possibility of an unrecognized fungal etiology in the setting of impaired ocular surface defense. It is worth noting that ocular function tests provided key insights into understanding the patient's susceptibility. The reduction in Schirmer's test, shortened BUT, and elevated tear osmolarity all indicated decreased tear film stability, which increased the likelihood of fungal adhesion. Additionally, the significant reduction in corneal nerve fiber density indicated decreased corneal sensitivity and impaired epithelial repair capacity, making early lesions harder to detect and more likely to spread deeper. Together, these findings support a clinically plausible pathway of “tear film abnormality → impaired epithelial defense → increased susceptibility to fungal invasion,” although this pathway remains inferential in the absence of direct mechanistic testing.

This study has several limitations. First, it is based on a single case and therefore cannot establish a causal relationship between immunosenescence and *Fusarium* keratitis. Second, no tear cytokine profiling, ocular surface immune assessment, histopathological examination, or molecular pathway analysis was performed. Therefore, immunosenescence should be interpreted only as a speculative, literature-supported explanation rather than direct evidence from this patient. Third, molecular sequencing was not performed. Although morphology and MALDI-TOF MS supported identification of the isolate as *F. solani* / FSSC, the precise species within the FSSC could not be definitively determined. Future prospective studies with larger sample sizes, direct immunological assessments, and molecular sequencing are needed to clarify both host susceptibility mechanisms and pathogen-level classification in fungal keratitis.

## Clinical trial

Not applicable

## Authors’ contributions

TT: Study design and manuscript writing. GD, YX, and XJ: Experimental execution and data analysis. HY: Clinical case data collection and analysis. TT and HY: Manuscript drafting and revision. All authors contributed to critical aspects of the study, reviewed the final manuscript, and approved the version submitted for publication.

## CRediT authorship contribution statement

**Huiyi Wu:** Conceptualization, Formal analysis. **Yuxing Gao:** Investigation. **Xi Jiang:** Methodology, Investigation, Formal analysis, Data curation. **huiyi wu:** Writing – review & editing, Formal analysis. **Guodong Liang:** Methodology, Investigation. **Tingting Huang:** Writing – review & editing, Writing – original draft, Formal analysis, Conceptualization.

## Publication consent statement

We, as the author(s) of the manuscript titled “Fusarium Keratitis in an Older Adult Without Exogenous or Traumatic Risk Factors: A Case Report on Intrinsic Ocular Surface Predisposition”, hereby voluntarily submit the manuscript to IDCases and authorize its publication in electronic format.

We confirm that the manuscript is an original work and has not been published or submitted elsewhere in any form. Furthermore, we certify that all listed authors have made significant contributions to the research and writing of the manuscript and have unanimously agreed to its submission and publication.

By signing below, we transfer the rights of publication, distribution, and reproduction of this manuscript in electronic format to IDCases, and we agree to abide by the journal’s policies and guidelines.

## Ethics approval and consent to participate

This study follows the ethical principles of the Declaration of Helsinki and has been approved by the Ethics Committee of Donghai Hospital Affiliated to Kangda College of Nanjing Medical University (Approval No.: LL2025–026).All participants provided signed informed consent.

## Funding

None.

## Declaration of Competing Interest

The authors declare that they have no known competing ffnancial interests or personal relationships that could have appeared to inffuence the work reported in this paper

## Data Availability

Not applicable
